# Evidence-informed decision-making by professionals working in addiction agencies serving women: a descriptive qualitative study

**DOI:** 10.1186/1747-597X-6-29

**Published:** 2011-11-07

**Authors:** Susan M Jack, Maureen Dobbins, Wendy Sword, Gabriela Novotna, Sandy Brooks, Ellen L Lipman, Alison Niccols

**Affiliations:** 1 School of Nursing, McMaster University, Hamilton, Ontario, Canada; 2 Offord Centre for Child Studies, Hamilton, Ontario, Canada; 3 Department of Psychiatry and Behavioural Neurosciences, McMaster University, Hamilton, Ontario, Canada

**Keywords:** Substance use, knowledge translation, evidence-informed decision-making, research utilization

## Abstract

**Background:**

Effective approaches to the prevention and treatment of substance abuse among mothers have been developed but not widely implemented. Implementation studies suggest that the adoption of evidence-based practices in the field of addictions remains low. There is a need, therefore, to better understand decision making processes in addiction agencies in order to develop more effective approaches to promote the translation of knowledge gained from addictions research into clinical practice.

**Methods:**

A descriptive qualitative study was conducted to explore: 1) the types and sources of evidence used to inform practice-related decisions within Canadian addiction agencies serving women; 2) how decision makers at different levels report using research evidence; and 3) factors that influence evidence-informed decision making. A purposeful sample of 26 decision-makers providing addiction treatment services to women completed in-depth qualitative interviews. Interview data were coded and analyzed using directed and summative content analysis strategies as well as constant comparison techniques.

**Results:**

Across all groups, individuals reported locating and using multiple types of evidence to inform decisions. Some decision-makers rely on their experiential knowledge of addiction and recovery in decision-making. Research evidence is often used directly in decision-making at program management and senior administrative levels. Information for decision-making is accessed from a range of sources, including web-based resources and experts in the field. Individual and organizational facilitators and barriers to using research evidence in decision making were identified.

**Conclusions:**

There is support at administrative levels for integrating EIDM in addiction agencies. Knowledge transfer and exchange strategies should be focussed towards program managers and administrators and include capacity building for locating, appraising and using research evidence, knowledge brokering, and for partnering with universities. Resources are required to maintain web-based databases of searchable evidence to facilitate access to research evidence. A need exists to address the perception that there is a paucity of research evidence available to inform program decisions. Finally, there is a need to consider how experiential knowledge influences decision-making and what guidance research evidence has to offer regarding the implementation of different treatment approaches within the field of addictions.

## Background

The treatment of substance abuse problems in women presents special challenges. Rates of substance abuse in women have been increasing [1] and research suggests that women are more vulnerable to the adverse physiological consequences associated with substance abuse than men [2]. Substance abuse in women is associated with a unique constellation of risk factors and needs, including greater prevalence of mental health problems, histories of physical or sexual abuse, serious medical problems, poor nutrition, relationship problems, exposure to intimate partner violence, and deficits in social support [3]. In addition, women dealing with substance abuse issues are often exposed to the stress associated with severe economic and social problems such as lack of affordable housing and homelessness [4]. Finally, the majority of women who abuse substances are of childbearing age [5] and maternal substance abuse has been associated with child maltreatment and poor long-term outcomes for children [6-8]. It is important, therefore, that effective interventions for this very vulnerable population are identified and implemented.

Promising models of integrated (or comprehensive) treatment programs that include on-site pregnancy-, parenting-, or child-related services with addiction services have been developed but not widely implemented, despite systematic reviews supporting their potential effectiveness [9-12]. Other systematic reviews and meta-analyses reveal a growing body of research evidence regarding effective approaches to the prevention and treatment of substance abuse [13]. Implementation studies suggest that the adoption of evidence-based practices in the field of addictions remains low [14-16]. There is a need, therefore, to better understand decision-making processes in addiction agencies in order to develop more effective approaches to promote the translation of knowledge gained from addiction research into clinical practice [14].

Among other health system stakeholders there is an emerging understanding that research evidence, if translated effectively, will lead to the provision of more effective programs and practices and subsequently improved health outcomes [17]. Evidence-informed decision making (EIDM) involves translation of the best available research evidence from a systematically collected, appraised, and analyzed body of knowledge, which is then considered alongside other forms of information to make program, practice or policy decisions [18]. While EIDM is a well known and an increasingly established concept in health care settings, it is a relatively new term in the addictions field [13,19].

Factors identified as impacting EIDM include individual decision makers, the system, patients, and research evidence [18]. At the individual decision-maker level, important factors include past professional or educational experiences, beliefs, values, and skills; at the system level, resources (both human and financial), legislation, protocols, and societal norms [17,20] are important factors. Commonly identified barriers to EIDM are lack of time, limited access to research evidence [20,21], limited capacity to appraise and translate research evidence, and a resistance to change traditional practices [22,23]. Specific to addiction treatment, an identified barrier to using empirically supported treatment models is personal experiences with addiction and recovery among clinical practitioners [24,25]. Furthermore, many addiction agencies exist as community-based organizations (CBO) working in parallel to traditional health systems. CBOs are typically grassroots or non-governmental agencies, which Wilson et al [26] define as organizations directed by missions and offering services that reflect the values of its constituent community and governed by an elected board that includes community members. Additionally, CBOs are funded through a combination of government and philanthropic resources, and organized to provide services and programs influenced specifically by the community's values and the CBO's mission [26]. The uniqueness of these types of organizations compared to clinical health settings may also influence the types of strategies required to move research evidence into local practice and policy [27].

In order to advance knowledge and the practice of EIDM in the field of women's substance abuse, a program of research known as Connections was established in Canada. The focus of this five-year program of research is the development and evaluation of innovative knowledge transfer and exchange strategies to facilitate EIDM among addiction agencies serving women [28]. For the purpose of this study, we have adopted Lomas et al's definition of evidence or "the notion of evidence concerns facts (actual or asserted) intended for use in support of a conclusion." [[29]; p.1]. However, Lomas et al [29] further conclude that different stakeholders hold different views of evidence. They suggest that researchers perceive it as knowledge gained through empirical methods and health system decision makers perceiving evidence more colloquially as any type of information that can be used to determine a fact or influence beliefs [29]. Therefore, a key component in developing tailored knowledge translation and exchange strategies for decision makers in addiction agencies serving women is to understand their perceptions of what constitutes evidence, as well as their knowledge, attitudes, and beliefs toward EIDM. The aim of this paper is to report the findings of one study within this larger research program. The objectives of this paper are to describe: 1) the types and sources of information used to inform practice-related decisions within Canadian addiction agencies serving women; 2) how decision makers at different levels (executive directors, program managers, service providers) report using research evidence in decision-making; and 3) individual and organizational factors that influence EIDM.

## Methods

The principles of fundamental qualitative description, as described by Sandelowski [30], guided all sampling, data collection, and analysis decisions. This qualitative approach facilitates the comprehensive exploration and description of phenomena being studied and provides researchers the opportunity to answer practical questions of relevance to service providers and other decision makers [30]. We obtained approval to conduct this study from the Hamilton Health Sciences/McMaster University Faculty of Health Sciences Research Ethics Board.

A purposeful sample of decision makers working in Canadian agencies providing addiction treatment services to women were invited to participate. A decision-maker was defined as any single individual within the organization who held formal or informal power to influence a treatment, service or program decision, including decisions related to planning, implementation, delivery, management, or evaluation. Based on past qualitative studies exploring the processes of research use by decision makers across different health fields [31,32], we estimated recruiting 20-30 participants to reach data saturation or the point in the study where no new data relevant to the primary study objectives were emerging. Study inclusion criteria included: 1) ability to speak English; and 2) employment in an agency providing addiction treatment services for women. We used maximum variation sampling [33] to recruit participants who varied on characteristics related to agency size, location across four geographic regions of Canada, type of program (integrated or non-integrated treatment services for women and children), and community type (urban or rural). To capture variation in the nature of decision-making experiences, we used stratified purposeful sampling [33] to ensure inclusion of participants classified as clinical (front-line service providers), managerial, and administrative decision makers.

Two snowball sampling strategies were used to identify the purposeful sample: 1) researchers with expertise in the field of addiction services for women and members of the Connections Advisory Committee identified potential key informants who could provide a rich and detailed description of the types of evidence used to inform addiction treatments and program decisions; and 2) at the end of each interview, study participants were asked to identify other individuals who met the study inclusion criteria. Recruitment of new study participants continued until the data analysts determined that no new information about: the types and sources of evidence used in decision-making, how research is used in decision-making, and factors influencing EIDM was emerging from the interviews.

Each study participant was invited to complete a single in-depth semi-structured telephone interview lasting approximately 60 to 90 minutes. The study was described in detail to each participant and informed consent to participate was obtained in writing. A semi-structured interview guide was developed based on Dobbins and colleagues' [34] theoretical framework for the dissemination and use of research evidence and included questions that explored: participants' definitions of evidence and EIDM; their perceptions of the types of evidence used within their agency; the processes to access and use research evidence in decision making; and the barriers and facilitators influencing access to, and the uptake of, research evidence within the agency. This interview guide was pilot tested with four clinicians with expertise in the field of addictions and experience working in front-line addiction agencies. Two research assistants with experience in qualitative data collection conducted the interviews with decision makers. In keeping with a hallmark of qualitative research, data collection and analysis occurred simultaneously. As new themes emerged in the analysis that required further explanation, the interview guide was revised accordingly. Permission to digitally record each interview was requested and the interviewers maintained detailed field notes documenting the context of interview, primary emerging themes, and new concepts to explore in subsequent interviews. As an honorarium for participation, each individual interviewed received a $25 gift card from a local bookstore.

All of the interviews were transcribed verbatim, with identifying information removed to ensure participant anonymity and confidentiality, and checked for accuracy. We used a directed content analysis approach [35] and used core concepts from Dobbins et al's theoretical framework [34] to identify an initial list of codes for data analysis. Early in the study process, two transcripts were individually reviewed and coded, and then discussed by research team members (SJ, MD, SB, EL, WS). Through this process, consensus was reached on all of the coding categories to be applied to the transcripts. Using these categories and the questions from the interview guide, we developed a structured codebook. Each transcript was coded line-by-line using NVivo 8.0 Software and the codes were subsequently collapsed into the broader categories. Using these categories, written summaries of each participant's interview were developed. Data were then synthesized by level of decision maker. We also used a summative approach to content analysis [35] to identify the most frequent types of evidence, sources of evidence, and factors influencing research use as identified by the different levels of decision makers interviewed for this study. The analytic strategy of constant comparison [36] was used to identify similarities and differences in experiences and perceptions of executive directors, program managers, and service providers. A written synthesis of the data was circulated to all study participants for the purpose of member checking to ensure that the research team's interpretation of the data was accurate.

The diversity of backgrounds and experiences among members of the research team contributed to data credibility and promoted data dependability, or the consistency of codes and findings emerging across interviews, during the analytic phase [37]. As a group, the investigators have clinical and research experience in the fields of maternal and child health, public health, nursing, psychology, and psychiatry. Several team members have experience evaluating knowledge transfer and exchange processes (SJ, MD, SB). Two team members (WS, AN) have experience evaluating integrated services for women with substance abuse issues and their children and one (AN) has clinical experience in this field.

## Findings

A purposeful sample of 26 decision-makers (eight executive directors, 12 program managers, and six service providers) recruited from 24 agencies providing addiction treatment services to women completed an in-depth qualitative telephone interview. Each participant was assigned a code and a number (i.e. Executive Directors - ED, Program Managers - PM and Service Providers - SP) to allow for better identification of the excerpts from the respective interviews. Of these individuals, 14 participants (three executive directors, seven program managers, and four service providers) completed the member checking process, which augmented and confirmed the research team's interpretations of the interview data. Participants were recruited from all ten Canadian provinces and one of three territories. Just under half of the participants indicated that they worked in agencies serving urban/rural populations, six served rural or remote populations, and five served urban populations. This purposeful sample was well positioned to provide in-depth descriptions about EIDM in the field of women's addiction services and to provide commentary about factors influencing research uptake as participants had, on average, 17 years of experiencing working in the field. Study participants had a range of educational backgrounds including addictions, education, medicine, or social work. However, all of the program managers had a professional background in social work. A summary of participant characteristics is presented in Table 1. In presenting the findings, the term "clients" is used to refer to women seeking services at substance use treatment agencies in order to be consistent with the language used and shared in the interviews.

**Table 1:** Participant Attributes

	Service Providers *n *= 6	Program Managers *n *= 12	Executive Directors *n *= 8	Total Sample *n *= 26
Gender				
Female	*n *= 6	*n *= 10	*n *= 6	*n *= 22
Male	-	*n *= 2	*n *= 2	*n *= 4

Education				
High School	-	*n *= 1	*n *= 1	*n *= 2
College	*n *= 1	-	-	*n *= 1
Bachelors	*n *= 2	*n *= 5	*n *= 2	*n *= 9
Masters	*n *= 3	*n *= 6	*n *= 5	*n *= 14

Mean years in current position	3.0 (Range 1-8)	7.2 (Range 1-30)	5.5 (Range 0.5-20)	5.5

Mean years working in addictions	13.3 (Range 3-29)	16.5 (Range 3.5-33)	21.0 (Range 1-39)	16.6

### Types of Evidence Used in Decision-Making

Across all three decision-maker groups, individuals reported locating and using multiple types of evidence to inform program or treatment decisions. The most common types of evidence used included: research evidence (results from longitudinal surveys, surveillance studies, qualitative research or intervention/prevention studies); best practice guidelines; perceived best practices (programs currently conducted in other locations and perceived to be ideal models of service provision); local program evaluations; client needs assessments; expert opinion; personal professional experiences; and an individual's personal experiences of addiction and recovery.

At the service-provider level, participants expressed a preference for relying on perceived best practices, information from client needs assessments, and their own professional experiences when making decisions. A majority of the service providers acknowledged that research evidence has been used within their agency to inform program decisions. However, at the individual level, there was significant variability in perceptions of the value of research evidence. At one end of the spectrum, some service providers identified that research evidence is important to integrate into decision-making as it creates the opportunity to implement programs that will have a known positive effect on client outcomes. Some participants also expressed the importance of ensuring that their clinical actions would not be potentially harmful to their clients, for as one service provider (addiction counsellor) shared:

It [research evidence] gives me a sense of assurance and credibility that what I'm doing is in the right direction. I mean I could be just going out there willy-nilly and waving magic wands and still get paid for it. But I want to know that what I'm doing is not going to be harmful to the people I am working with (SP011).

Conversely, some service providers were more likely to place a greater value on personal and professional experiences, particularly if the research evidence contradicts "what is known to be best for the client" based on their experiences of working in the field. One counsellor with over 25 years of clinical experience expressed that:

I hear my supervisor talk about best practices a lot, but the truth of the matter is - I'm going to be very honest with you - I don't really pay a lot of attention to it. Just based on my own experiences, the abstinence-based twelve-step philosophy is what works (SP007).

Furthermore, some service providers gave examples of specifically locating research evidence to justify their choice of treatment model (most commonly an abstinence-based, 12-step treatment approach), to endorse their experiential knowledge or to validate their current professional practices.

All of the service providers interviewed perceived that research evidence in the field of addictions is outdated or not relevant to their specific client populations, or that there is a gap between what researchers study and what information would be of practical value for them.

Of the 12 program managers interviewed, ten managers identified expert opinion as a key type of evidence carrying significant weight in influencing their decisions. These participants identified that if a particular program or intervention was endorsed or recommended as a best practice by an individual or agency recognized as an "expert" (e.g. Health Canada, Canadian Centre for Substance Abuse, Addictions Ontario, Centre for Addiction and Mental Health) or perceived to be highly credible in the field, the likelihood of adopting the recommended program or intervention locally would increase.

### Use of Research Evidence

The majority of program managers and all executive directors indicated that they locate and use research evidence in their decision making, and that greater priority is placed on using research evidence and best practice guidelines to inform decisions than experiential knowledge. As one program manager noted:

I think quite frankly that if we're not actually using research to inform decisions, then we assume that we are on the right path and that we're doing things that are helpful to our client population - which may in fact not be [helpful] (PM008).

Participants from these two groups provided examples of using research evidence to: select an intervention or program to implement within the organization; identify trends and emerging issues in the field; develop background information on an issue for a report, presentation or grant proposal; gain new perspectives about potential strategies for addressing issues presented by women seeking service; and to endorse experiential knowledge. As one executive director explained:

I'm heavily guided by research evidence. That's how I write my proposals. And it's just a way of being credible and saying that, 'Well, this is an approach that works and this is why we want to offer this program. (ED017)

Furthermore, there was consensus among all levels of decision makers that once valid research evidence is located it: must be validated by program staff and clients as relevant; generally must be adapted to meet the local needs of the community and the agency's community partners before the evidence-based intervention or program can be implemented; and is only one of many types of information considered in the decision-making process.

When making a decision about the types of programs, interventions or treatment modalities to use, decision makers balance the results of research studies with information about: their agency; the service providers employed by the agency; the women served by the agency; the broader social and political context in which addiction services are provided; existing programs offered by their community partners; and characteristics of the research evidence under consideration. The attributes related to each influencing factor that are considered in the decision-making process are summarized in Table 2. Across all decision-maker levels, consensus emerged that the "ideal" intervention or program with the greatest chance of being implemented within an agency would be: 1) evidence-based; 2) match the treatment philosophy of both the agency and the service providers in the agency (e.g., based on either an abstinence-focused, 12-step program or a harm reduction model); 3) have been successfully implemented in another agency; 4) be supported by credible experts in the field; 5) be seen to meet the expressed needs of women seeking services; 6) be supported by community partners; and 7) require minimal financial or human resources to implement. No one type of knowledge, including research evidence, was considered to have greater relevance or impact on the adoption process compared to the other types of knowledge.

**Table 2:** Factors That Influence Decision-Makers' Capacity To Implement Evidence-Informed Programs or Interventions

Influencing Factor	Characteristics
Social or political mandates	• Requirement by funding source(s) to implement evidenced-based programs • Provincial requirements to implement or provide specific programs or services

Agency mandate and resources	• Agency vision and mission statement • Treatment philosophy endorsed by agency • Available human and financial resources

Community	• Decision-makers seek to avoid a duplication of services within a community • Observed successful implementations of programs or services by community partners • New program or service complements existing services within the community

Agency service providers	• Personal values and beliefs • Professional expertise • Personal experiences of addiction and recovery

Agency clients	• Cultural expectations • Identified needs and preferences for services

Research evidence	• Validity of the study results • Generalizability of the study results to the local context • Evidence of positive client outcomes • Successful implementation of the evidence (e.g. an evidence-based intervention) • Evidence-based program or intervention endorsed by a credible expert or agency • Cost required to implement the evidence-based program or intervention

Across interviews there was significant discussion about the role of individual service providers' personal experiences with addiction and recovery in informing treatment and program decision-making. Participants explained that many individuals who work in addictions have personal knowledge of the process of addiction and recovery, either from their own experiences or that of a friend or family member. However, there appears to be a tension in the field around if, and how, this experiential knowledge should be used in decision-making. Overall, study participants acknowledged that experiential knowledge could contribute to the development of mutuality and trust between the service provider and client. The small number of study participants who disclosed a personal history of addiction and recovery during the interviews reported that their experiences significantly influence their professional and clinical decisions. One clinician (SP007), in recovery from alcohol and crack addiction, acknowledged making decisions based on "what I feel is right, which is my own experience." Many program managers and executive directors identified the importance of validating counsellors' lived experiences, but highlighted the potential risks to relying solely on experiential knowledge to inform decisions.

One of the risks noted is that service providers may reject evidence-based practices in favour of their personal experiences. One executive director (ED017) explained that some counsellors have the attitude that, "I've been through it [addiction and recovery] and I know all about it. I don't need to know about anything the research says 'cause what do researchers know?" There also was a belief that if a service provider chooses to disclose too much experiential knowledge, there is a risk of crossing therapeutic boundaries with the client. As one program manager (PM013) stated, "When you're trying to help a client, your job as a counsellor isn't to tell them what worked for you, but help them find what will work for them." The majority of participants concurred that experiential knowledge should not be the primary source of evidence for making agency-wide decisions regarding treatment models or interventions but recommended that it is useful in providing a deeper understanding of the issues individuals with addictions experience and for adapting best practices or research to meet the needs of women seeking services. There also was general agreement that when both experiential evidence and research evidence are taken into consideration in decision making that a more holistic understanding of the issues related to substance use and treatment is realized.

### Sources of Evidence

Study participants reported searching for and accessing evidence for decision-making across a wide variety of sources. The most common sources included websites and from colleagues with perceived expertise in substance abuse prevention and treatment. The majority of participants identified locating information from freely accessible websites hosted by agencies affiliated or funded through provincial or federal organizations that provide access to evidence-based resources. These resources included registries of best-practices, (e.g., British Columbia Ministry of Health Services, Best Practices in Mental Health and Addictions, Public Health Agency of Canada Best Practices Portal), general information about drug prevention and treatment (e.g., Health Canada, Canadian Centre on Substance Abuse), and summaries of research evidence. One service provider (SP011), in describing use of the Health Canada website, commented that, "We as Canadians have an implicit trust that most of the information provided to us from a federal agency is going to be supported in research and fairly accurate."

Participants also regularly access websites hosted by non-profit charitable associations (e.g., Addictions Ontario) that provide information about community resources, disseminate key reports on issues such as mental health or addictions, host communities of practice, and provide links to other resources. Approximately half of the participants identified that they often conduct broad, general "Google" searches to locate websites where they can freely access evidence and resources. Through these Internet searches, a small number of participants explained that they had found and subsequently used information from websites of independent companies, most often psychologists offering workshops or training programs for counsellors. The majority of service providers and a small number of program managers and executive directors also spoke about the value of being connected to electronic mailing lists that regularly send them updates about research and information emerging in the field of addictions and substance use or being a part of a virtual community of practice (e.g., Coalescing on Women and Substance Use virtual discussions through the British Columbia Centre of Excellence for Women's Health).

All of the service providers and just over half of the program managers and executive directors identified other individuals as a primary source of their information for decision-making. Service providers generally spoke about seeking information from colleagues working within their agency or community partners because their professional expertise was valued and their philosophies around treatment (abstinence-based vs. harm reduction) were similar. Program managers and executive directors sought information from experts, who were likely to be administrative colleagues in other agencies, researchers, or individuals from agencies with known credibility in the field. Across all levels of decision makers, study participants reported accessing research evidence through peer-reviewed journals but very few accessed research evidence in the form of systematic reviews through the Cochrane Collaboration.

### Factors Influencing Research Use in Decision-Making

Study participants were asked to identify both barriers and facilitating factors to using research evidence in their decision-making. The most common barriers to using research evidence in decision making were: lack of time; a work environment with competing priorities; and the perceived gap between research evidence and practice. Decision makers highlighted specifically a lack of time to search the Internet or databases for evidence, read information or reports, critically appraise and summarize research reports, and compare findings across studies. Decision makers across all levels also described working within a field where the majority of agency resources and funds are directed towards service delivery, treatment, and educational activities and where there is little mandate to allocate resources toward conducting research or integrating research into practice. Without a mandate to focus on evidence-informed practice or policy, the decision makers identified that they then prioritize service delivery over any activities related to EIDM. Decision makers were highly critical of the current base of evidence in the field of addictions and its relevance to practice, citing that research is outdated and that it is difficult for researchers to keep up with trends emerging at the practice level. Decision makers also cited a significant gap between what researchers highlight as important to study and what practitioners identify as current practice issues requiring evaluation.

One barrier that was unique to service providers was the perceived lack of interest or resistance to adopt evidence-informed interventions. One program manager (PM004) explained that:

One of the interesting anomalies...in addictions services is the whole resistance to change. You know, the whole idea of 'if it ain't broke, don't fix it.' Sometimes though, it was always broke and you have to point that out [to staff] and say, 'You know, what you have been doing for the last 15 years, although it made you feel good, unfortunately has not really been effective with this particular population.'

Executive directors and some program managers explained that front-line staff may be resistant to adopt evidence-based strategies when the research evidence contradicts their current professional practices or when they are put in a position to reflect on, and potentially change, their current approaches to working with clients. One executive director explained:

They [front-line staff] 'know what they know.' And they don't want what they know challenged by new information. You have to acknowledge that that's a fact. People have their beliefs, they know what they know and they don't want to argue about it, they want to apply it. It's a resistance (ED015).

Decision makers identified four primary facilitators to increasing the use of research evidence in practice. First, the board of directors and senior administration must support the philosophy of evidence-informed practice and provide supports for front-line staff and managers to have access to research evidence, to develop the skills required to appraise and apply evidence, and to be actively involved in primary research activities. Secondly, research is more likely to be reviewed for potential adoption if there is an individual within the agency who has the formal responsibility and skills to search for, retrieve, appraise, summarize, and interpret the research evidence for all levels of decision-makers. One executive director (ED015) explained, "To make good information palatable, often you need an emissary." This individual would also be responsible for identifying strategies for communicating the results of studies and disseminating key messages across the agency. The participant (ED015) further suggested, "You need a go-to person in each area that would talk to you and say, 'Here's some really good stuff that's coming by lately, have a look at it." Third, it was identified that research evidence is more likely to be adopted if it is translated into plain language and is accompanied by recommendations on "how-to" apply the findings into practice. The final facilitating factor was an endorsement of establishing formal partnerships between universities and addiction agencies. It was highlighted that these partnerships would create opportunities for researchers to answer practice-informed research questions and for agency staff to have access to current research evidence and individuals skilled in interpreting research jargon. One program manager concluded that:

If we had partnerships with universities, right, where it would be possible to call up and say, "Okay, so this is what we're looking at. Can you just spend fifteen minutes of your time and just tell me what you know or where I can go for this information?" It'd be really helpful to have those kinds of partnerships (PM019).

## Discussion

Within the field of addiction services for pregnant women and mothers, this is the first Canadian study to explore EIDM among service providers, program managers, and executive directors and their perceptions of what, and how, evidence is used in program decision making. Across all levels of decision making, participants consistently reported locating and using multiple types of evidence to inform treatment choices and program options for their clients. It is important to note that within these predominantly CBOs, that there is a preference for relying on locally collected information to inform decision-making processes. While research evidence is acknowledged as an important source of information, it is used in varying ways by the different levels of decision-makers. To locate evidence to inform decisions, study participants spoke to their frequent use of web-based resources or by engaging with individuals with perceived expertise in the field. Finally, decision-makers identified four key activities that are required to promote the uptake of research evidence in their agencies: 1) administrative support for creating a culture that fosters EIDM; 2) locating knowledge brokers within the agency; 3) transforming research evidence into more accessible formats; and 4) establishing partnerships and engaging with university-based academics. The findings from our study reflect similar attributes as outlined in Wilson et al's framework [26] for community-based knowledge transfer and exchange strategies and endorse the need for developing KTE strategies unique to this context.

Specific to women's addiction services, service providers reported that their clients' perspectives were important considerations in decision making about practice [38-40]. While participants indicated that they value research evidence in decision making, greater emphasis is placed on considering data from program evaluations, reports of local or international best practices, client preferences, experiential knowledge, professional judgment, and the recommendations of perceived experts in the field. Kothari and Armstrong [27] suggest that within CBOs, some decision-makers have a preference for, and use, data collected locally because it is so highly relevant to the local context in comparison to the perceived generalizability of published research findings.

Research evidence is often described as being used by decision makers in three different ways: instrumentally, conceptually or symbolically [41,42]. The instrumental use of research evidence refers to the direct use of research findings to inform a decision whereas the conceptual utilization of research evidence refers to a process of enlightenment whereby findings from a study provide decision makers with a new perspective or insights about a phenomenon [42]. Symbolic utilization is when decision makers purposefully seek out research evidence to validate a program, treatment or policy decision that has already been pre-determined [41]. Unique differences in how research evidence is used in decision-making were observed between service providers and the more senior levels of decision makers, which included the executive directors and program managers. The latter group was more likely to identify examples of using research instrumentally to inform key decisions around the type of programming to adopt and implement within the agency. It is important to note, however, that after accessing research evidence about effective interventions or programs, decision makers would then locally adapt the intervention to meet the needs of the local agency and clients. When evidence-informed interventions are adapted and not implemented with fidelity to the models originally evaluated, agencies may expect to see a reduction in the impact of the program on desired client outcomes. However, strategic approaches to guide decision makers in accessing, evaluating and adapting evidence-informed interventions for integration into practice have been developed in public health [43]. Application of these strategies may be beneficial within the field of addictions. Kothari and Armstrong [27] argue that supporting decision-makers within CBOs to adapt research evidence to their local context is a key strategy for advancing EIDM in the field.

Program managers and executive directors also use research conceptually, citing examples of locating surveillance data to identify emerging trends in the field and reviewing research evidence to gain a new perspective or understanding of the issues experienced by women seeking treatment for substance abuse. In comparison, service providers reported that they did not directly use research evidence instrumentally but were more likely to provide examples of using it symbolically; they often used this type of evidence to validate their current practices or, for some, their personal experiential knowledge of addiction and recovery. The extent to which symbolic use of evidence occurs in the field is problematic and warrants further investigation. Ongoing use of research solely to validate existing practices, particularly when practices are informed by experiential knowledge, may extend the delivery of interventions with less than ideal effect on client outcomes and serve as a barrier to the development and integration of programs, services, and interventions that are known to have positive effects on client outcomes

The degree to which personal experiential knowledge of addiction and recovery is valued and used by some front-line addiction counsellors is a unique finding compared to similar studies we have conducted in the fields of child welfare [32], public health [20], and environmental health [31]. Our findings suggest there is a tension in the field regarding the use of personal experiential knowledge to inform decision-making and the appropriate contexts in which to use this knowledge. There was recognition across decision-maker groups that this type of knowledge has the potential to enhance understanding of clients' recovery experiences and that it is important to find strategies for integrating findings from experiential knowledge with scientific evidence.

Enkin and Jadad [44] suggest that when experiential or anecdotal knowledge and scientific evidence support the same conclusions, there is a validation of this knowledge and the potential impact on decision-making is likely considerable. However, these authors identify a potential for conflict in decision-making when scientific findings are contrary to the beliefs held by decision makers. Indeed, studies on organizational change in human services organizations suggest that to successfully implement evidence-informed practices, professionals responsible for disseminating innovations have to challenge underlying assumptions and beliefs relative to "old" treatment practices. It is only when those established norms and practices weaken or dissipate that new models can be implemented [45,46].

In this qualitative study, many decision makers highlighted the influence of an organization's treatment philosophy (generally a 12-step abstinence approach or a harm reduction model) on decision-making. The findings suggest an inherent tension faced by senior decision makers working in the field of addictions who must select a treatment philosophy and treatment interventions to implement within their organizations. The tension stems in part from the strong beliefs held by some regarding the effectiveness of abstinence programs compared to harm reduction models for the treatment of addictions. These firmly entrenched beliefs contradict the current best available scientific evidence on the effectiveness of 12-step approaches to influence important client outcomes. In a systematic review of eight trials (n = 3417), Alcoholics Anonymous or similar 12-step approaches were not effective in reducing alcohol dependence or other alcohol-related problems [47]. Therefore, it is important for senior decision makers to be sensitive to the potential for conflict when advocating for the adoption of an evidence-informed treatment that potentially contradicts service providers' beliefs or organizational philosophy.

Findings related to the attitudes about addiction and recovery held by clinical personnel with experiential knowledge are consistent with those of other studies that found that clinicians in recovery who endorsed a 12-step abstinence model were less likely to support and utilize evidence-informed motivational and behavioral treatment approaches [24,25,48]. These findings suggest that decision makers in addiction treatment services might be a more heterogeneous and complex group than in other service settings. Strategies to address the tension related to differences in treatment philosophies might benefit from the empirical research informed by institutional theory on the need to challenge and dissipate "old" concepts and norms that are incongruent with the values and norms of the innovative practices to be implemented [45,49]. The importance of addressing the role of ideological differences in EIDM has been accentuated by the findings of the survey of Canadian Centre for Substance Abuse (CCSA) of workforce in addiction services in Canada. This survey found that 28% to 35% of executive directors/agency heads in Saskatchewan, British Columbia, North West Territories, and Quebec identified having a personal history of alcohol or drug problems, with smaller percentages reported in Ontario (17%) and Atlantic Canada (5%) [50].

In addition to the types of evidence used to inform decision making, this qualitative study provided insight into the sources of information. Given that the majority of funding for addiction agencies is directed toward service delivery, it was not surprising to learn that decision makers search for and locate a preponderance of their information through unstructured Google searches or by accessing websites sponsored by provincial or federal government agencies that provide free access to a range of addiction-related resources. As such, ongoing support should be provided and maintained for organizations that have the mandate, capacity, and skill to host Internet sites that contain databases of searchable evidence-informed resources relevant to addictions. Given the preference for accessing resources through these types of websites, and the acknowledgment that the decision-makers highly value programs or interventions endorsed by credible organizations, it is imperative that posted recommendations be based on the current, best available evidence such as that found in systematic reviews. This type of approach has been used in the public health sector (e.g., healthevidence.ca) to support organizations in having equitable access to high-quality evidence from a reliable agency. It reduces the need for small organizations, with limited budgets, to invest locally in purchasing subscriptions to journals and other resources related to obtaining and appraising research.

In addition to identifying credible sources of information on the Internet, study participants expressed a preference for seeking information for decision-making or practice recommendations from individuals and organizations with perceived expertise in the field of treating women with addictions. Flodgren and colleagues [51] conducted a systematic review to assess the effectiveness of the use of local opinion leaders in improving professional practice and patient outcomes in acute care and primary care practices. They concluded that the integration of opinion leaders, alone or with other interventions for promoting the uptake of research evidence into practice has the potential to successfully influence the uptake of evidence-informed practice. Given this premise, the identification and use of local opinion leaders to influence the adoption of treatment programs and interventions with demonstrated effectiveness may hold significant promise in the context of women's addiction services.

Senior decision makers shared that having the opportunity to observe the successful implementation of an intervention in another organization is a factor that would positively influence their decision to adopt the treatment or program. In his diffusion of innovations theory, Rogers [52] identified five specific attributes that influence the adoption of any innovation, one of which is observability or the degree to which the impact of a specific innovation can be observed by others. Given the value that decision makers place on knowing that other agencies or organizations have successfully implemented an evidence-informed intervention should provide an incentive for researchers in the field of addictions to prioritize conducting, publishing, and widely disseminating results of program evaluations.

In this study, we also explored barriers and facilitators that influence the use of research evidence in decision-making. We found that the experiences of decision makers working in agencies serving women with addictions are largely congruent with the experiences of decision makers in various health care settings [e.g. [20,53]], social services [e.g. [54]], and child welfare [32]. Barriers and facilitators are fairly consistent across different levels of decision makers. One exception that emerged was service providers' (but not administrators') resistance to adopting research evidence and their lack of interest in research. Dixon [55] identified that the introduction of evidence-informed practice into addiction agencies is likely to create feelings of resistance and requires senior decision makers to develop expertise in managing change at both system and individual staff levels.

Given that administrators are more likely to adopt EIDM than service providers, strategies could consider program managers and executive directors as potential change agents within addictions agencies. Findings by Dobbins et al. [20] support the need for a champion within agencies to coordinate action and to motivate and challenge others to think and practice in an evidence-informed way. A randomized controlled trial evaluating the impact of a knowledge broker (someone external to the organization who works one-on-one with individuals and teams within the agency to develop knowledge, attitudes, skill and culture for EIDM) found that this intervention had a significant influence on EIDM among public health departments that rated themselves as very low at baseline in the use of research evidence for program planning [56]. In another study evaluating a 'train-the-trainer' approach to knowledge brokering in centres providing services for children with disabilities, the training of knowledge brokers internal to the agency, who also received mentoring and support from an experienced knowledge broker, resulted in statistically significant improvements in the use of evidence-informed functional assessment tools by clinicians [57]. In this latter study, participants indicated that having someone internal who could access research evidence, appraise it, and interpret it for them was instrumental in facilitating the uptake of this knowledge into practice. We therefore recommend that there is a need for ongoing capacity development among program managers to train them on how to efficiently locate, appraise and then implement research evidence in decision-making.

Additionally, opportunities exist for capacity development within the academic arena. With increased emphasis being placed on the establishment of academic-decision maker partnerships to facilitate both the production and uptake of research evidence [17,26,27], increased responsibility is placed on applied health researchers to seek out opportunities for collaboration and identify research questions to answer timely and relevant practice and policy questions. However, to build on their credibility as important messengers for disseminating and interpreting research evidence [17], there is a responsibility for researchers to develop their skills in translating and communicating research findings.

As part of any given knowledge transfer and exchange strategy, in addition to identifying the type of information to disseminate, researchers have a responsibility to identify the most appropriate target audience to whom the research information is transferred [17]. It is imperative in developing tailored knowledge transfer and exchange strategies that research evidence is directed to individuals within the organization who have the autonomy, power, and resources to instrumentally influence, or to make recommendations about, treatment options or programs. Within the context of agencies providing addiction services to women, findings from this study indicate that it is prudent to invest in targeting decision makers at the manager level or higher. In addition, given that study participants perceived individuals with known expertise in the addictions field to be credible and important sources of information, it might be a valuable investment of knowledge transfer and exchange resources to target efforts towards known experts who could act as opinion leaders or champions. Organizations that were identified by the research participants as leading organizations (e.g. Canadian Centre for Substance Abuse and CAMH) have mandates and capacities to provide expert opinion and participate in the dissemination of research evidence to enhance the development of the field of addictions in Canada.

To promote the overall trustworthiness [37] of the findings from this qualitative study, several methodological strengths should be noted. Overall data credibility was enhanced through the use of member checking, data source triangulation, and researcher credibility. Data dependability was promoted through engaging multiple research team members in coding and analysis. An audit trail documenting study processes and decisions was also maintained. It is feasible to conclude that the findings from this study have transferability to other community-based Canadian addiction agencies serving women. Our findings, in particular the findings illustrating the factors influencing EIDM, reflect elements similar to the evidence-informed framework developed in medicine [58] and subsequently adapted for nursing [59], public health [60], and social work [61]. Therefore, the findings may be transferable and of relevance to decision-makers working in any health or social service field where there is a current culture of moving research evidence into practice and policy. One exceptional finding that may be specific to the field of addictions however, is the influence of experiential knowledge in the decision-making process. However, there are some study limitations that reduce the potential for transferability of these study findings. First, through the use of snowball sampling to recruit study participants there is a potential that the referred individuals hold similar values or beliefs to the study participants, thus potentially limiting our ability to explore the full breadth of experiences. Second, there was an unequal distribution of the sample across the three levels of decision-makers, with only six participants in the service provider group. Given the heterogeneous nature of the types of service providers delivering front-line care to women with substance use issues, further study of this unique group is warranted. Finally, given the qualitative nature of the study, it must be acknowledged that this is self-reported data, with a specific focus on individual perceptions and opportunities to directly observe decision-making processes was not embedded into the study.

## Conclusions

There is support among senior administrators in addiction agencies for integrating EIDM into practice. As a result, future knowledge transfer and exchange efforts should, in part, focus on targeting this specific group. Initiatives aimed at increasing the use of EIDM also should take into consideration the different value EIDM might have in the context of addiction services and particularly how EIDM can gain and maintain legitimacy among service providers. It has been argued that when the value of research evidence is low or inconsistent with the existing belief systems of professionals working in the field, the merit of available research evidence will likely remain unrecognized or contested [44]. This leads to under-utilization, solely symbolic use, or the complete disregard of research evidence [46,62].

It also is evident that researchers and decision makers focused on advancing EIDM in the field of addictions services for women need to consider the socio-political challenges of the opposing treatment philosophies of the 12-step abstinence versus harm reduction programs, and what the research evidence offers as guidance for either approach. Another important priority is for the field to address the perception that available research evidence is not relevant and does not address the needs of front-line decision makers. Ongoing support for community-university collaborations to plan and conduct practice-relevant research is required and desired.

Given that approximately one third of substance abusers are women of childbearing age [5], substance use among pregnant and parenting women is a serious problem for the child welfare system and a major public health concern. As the burden of suffering due to maternal substance abuse is great, the findings from this study are noteworthy and support the need for focused knowledge translation efforts in addiction agencies serving women. The effectiveness of these efforts warrants investigation, as the implications of widespread implementation of EIDM may include reduced costs to taxpayers, increased access, and more positive outcomes for mothers and children.

## Competing interests

The authors declare that they have no competing interests.

## Authors' contributions

SJ, MD, SB, WS and AN contributed to the development of the study design. Sampling and data collection procedures were led by SB, SJ and WS. SB completed the initial level of data coding, SJ completed the second level of data coding and developed the data summaries and syntheses. SJ, MD, SB, WS, AN and EL participated in analytic procedures to increase data dependability. SJ wrote the first draft of the manuscript with contributions from MD and GN. All authors contributed to and have approved the final manuscript.
